# Balancing revenue generation with capacity generation: case distribution, financial impact and hospital capacity changes from cancelling or resuming elective surgeries in the US during COVID-19

**DOI:** 10.1186/s12913-020-05975-z

**Published:** 2020-12-03

**Authors:** Joseph E. Tonna, Heidi A. Hanson, Jessica N. Cohan, Marta L. McCrum, Joshua J. Horns, Benjamin S. Brooke, Rupam Das, Brenna C. Kelly, Alexander John Campbell, James Hotaling

**Affiliations:** 1 Surgical Population Analytic Research Core (SPARC), Department of Surgery, University of Utah Health, Salt Lake City, UT USA; 2 Department of Population Health Sciences, University of Utah Health, Salt Lake City, UT USA

**Keywords:** COVID-19 pandemic, Critical care capacity, Resource allocation, Available hospital beds, Overcapacity

## Abstract

**Background:**

To increase bed capacity and resources, hospitals have postponed elective surgeries, although the financial impact of this decision is unknown. We sought to report elective surgical case distribution, associated gross hospital revenue and regional hospital and intensive care unit (ICU) bed capacity as elective surgical cases are cancelled and then resumed under simulated trends of COVID-19 incidence.

**Methods:**

A retrospective, cohort analysis was performed using insurance claims from 161 million enrollees from the MarketScan database from January 1, 2008 to December 31, 2017. COVID-19 cases were calculated using Institute for Health Metrics and Evaluation models. Centers for Disease Control (CDC) reports on the number of hospitalized and intensive care patients by age estimated the number of cases seen in the ICU, the reduction in elective surgeries and the financial impact of this from historic claims data, using a denominator of all inpatient revenue and outpatient surgeries.

**Results:**

Assuming 5% infection prevalence, cancelling all elective procedures decreases ICU overcapacity from 160 to 130%, but these elective surgical cases contribute 78% (IQR 74, 80) (1.1 trillion (T) US dollars) to inpatient hospital plus outpatient surgical gross revenue per year. Musculoskeletal, circulatory and digestive category elective surgical cases compose 33% ($447B) of total revenue.

**Conclusions:**

Procedures involving the musculoskeletal, cardiovascular and digestive system account for the largest loss of hospital gross revenue when elective surgery is postponed. As hospital bed capacity increases following the COVID-19 pandemic, restoring volume of these elective cases will help maintain revenue. In these estimates, adopting universal masking would help to avoid overcapacity in all states.

## Background

The novel Coronavirus Infectious Disease 2019 (COVID-19) is a highly transmittable virus that has resulted in over 16 million infections worldwide [1]. United States (US) health systems are designed for stable, predictable utilization patterns; they are generally unprepared for surge medical need as is required with the COVID-19 pandemic. Surge situations have historically been addressed by deploying temporary medical teams and tent facilities, or have overwhelmed the available resources with resultant excess morbidity and mortality [2]. Current estimates suggest at least 5% of the US population will contract COVID-19, of whom 15% will require hospitalization, and 5% will require intensive care [3–5]. At this rate, there will be 5 infected patients requiring hospitalization for every existing US hospital bed in addition to concomitant hospitalized non-COVID patients [6].

In order to decrease the resources needed during the peak of COVID-19, many countries have adopted physical distancing to attempt to flatten the curve of COVID-19 [7]. Some nations, such as China, have taken extreme measures such as forced quarantines and a complete shutdown of all aspects of society whereas other countries such as Sweden have chosen to remain largely open. The United States has emphasized physical distancing and suspension of non-essential business operations which have, until now, included elective surgical procedures. This has resulted in hospitals in areas with relatively few COVID-19 patients, such as Utah, functioning at only 30% of their typical inpatient and surgical capacity. The financial implications of cancelling elective surgical procedures has been devastating for healthcare systems with some systems currently losing upwards of $25 million USD per week [8].

However, to mitigate large anticipated surges in hospitalizations and deaths over the coming months, US hospitals need to sustainably absorb increases in patients requiring hospitalization and intensive care, while continuing to care for non-COVID patients. After a statement from the American College of Surgeons (ACS), many hospitals across the country ceased elective surgery to free beds and limit patient exposure. Recent models have suggested that the surgical backlog from these cancellations will be significant [9]. While cancelling elective surgical cases has increased capacity, the financial impact of these surgeries, including relative financial contribution by case type, has not been described. Furthermore, the epidemiology of the deferred elective surgical cases, case counts, and their resultant contribution to bed capacity has not been described.

We estimate the financial implications of cancelling elective surgeries using a national insurance claim database, Truven MarketScan (MS). This information can be used to inform strategies for resuming elective surgical procedures. We describe regional differences in capacity of US hospitals to absorb COVID-related inpatient surges if measures are not taken to slow the spread, describe elective case distribution, and present the relative financial impact and case counts of resumption of elective surgical cases.

## Methods

Our analysis is reported according to the Strengthening the Reporting of Observational Studies in Epidemiology (STROBE) Guidelines [10]. This study did not meet criteria for review by the Institutional Review Board at the University of Utah, Salt Lake City, Utah, USA according to United States law under the Health Insurance Portability and Accountability Act (HIPAA) as all data was fully de-identified.

### Data source and study population

Gross inpatient hospital revenue, outpatient surgical revenue, and inpatient and ICU (I/ICU) beds were estimated using Truven MarketScan (MS). MS billing data captured 8–16% of the entire US population from 2013 to 2017. For every MS patient, we determined the total number of I/ICU days from 2008 to 2017 for 161 million (M) enrollees and aggregated visit counts by month, state, and major diagnostic category (MDC). A person who switches insurance providers will appear serially in MS with two different IDs, thus the overall population is likely to be slightly smaller. Elective surgery admissions were identified by surgery revenue codes not associated with emergency revenue or provider codes. Surgical admissions were based on the presence of a surgical revenue code (REVCODE = 0360, 0361, 0362, 0367, 0369, 0975) or admission type (ADMTYP = 1) associated with the admission. Admissions occurring within 30 days post-elective (including outpatient) surgery were considered complications and categorized as resulting from elective surgery. Gross revenue was aggregated by MDC codes and surgical (elective vs non-elective) vs non-surgical. Analysis by MDC excluded eye, human immunodeficiency virus, health status and missing categories.

### Study variables and outcomes

MS data were used to report gross hospital revenue derived from inpatient admissions and outpatient surgeries for each MDC category by elective vs non-elective surgical and non-surgical. We calculated proportion of gross revenue from elective surgeries by state, and the proportion of I/ICU beds unoccupied, and occupied by elective and emergent cases by state. The average number of unoccupied beds was defined as the difference between the average number of I/ICU days and the maximum number of I/ICU days in a given state. We used data from the Harvard Global Health Institute (HGHI) to identify the total number of I/ICU patient beds per state and applied the proportion from the MS data to obtain estimates of occupied beds [11].

The Centers for Disease Control (CDC) Morbidity and Mortality Weekly Report (MMWR) was used to calculate the number of expected I/ICU beds occupied by age group [12]. We applied these age-specific rates to 5% of each state’s population using 2018 U.S. Census Bureau age-specific population estimates to calculate the number of expected hospitalizations and inpatient cases by state [13]. We calculated lower bound (LB) and upper bound (UB) estimates based on the CDC’s reported uncertainty.

### Statistical analysis

In order to estimate the financial implications of cancelling or resuming elective surgical cases, we calculated the distribution of gross revenue by MDC code and then by elective and non-elective surgical, and non-surgical classifications. To inform the balance of revenue and capacity generation, we illustrate the effect of cancellation of elective surgical cases on hospital capacity using the projections from the Institute for Health Metrics and Evaluation [14]. The models incorporate data on observed COVID-19 deaths, hospitalizations, and cases and are updated daily. Three scenarios are estimated: 1) The current projection assuming social distancing when deaths are higher than 0.8 per 100,000 people, 2) Continued easing of social distancing mandates, and 3) Universal Masks (95% use in public). We assumed the incidence density curves were single modal, with the highest peak being the first spike in cases. We assumed that patients occupied a hospital or ICU bed for an average of 9 days. Capacity was calculated as the difference between bed occupancy at the state level during the highest peak of cases and the number of hospital or ICU beds in the state.

We illustrated gross revenue by MDC code and by classification. We calculated the number of hospitalized patients in each state during the simulated pandemic peak using 11-day inpatient and 9-day ICU length of stay [4, 5, 15]. We then calculated the ratio of cases to total number of available beds per state with and without removal of elective surgery.

## Results

**Fig. 1:**
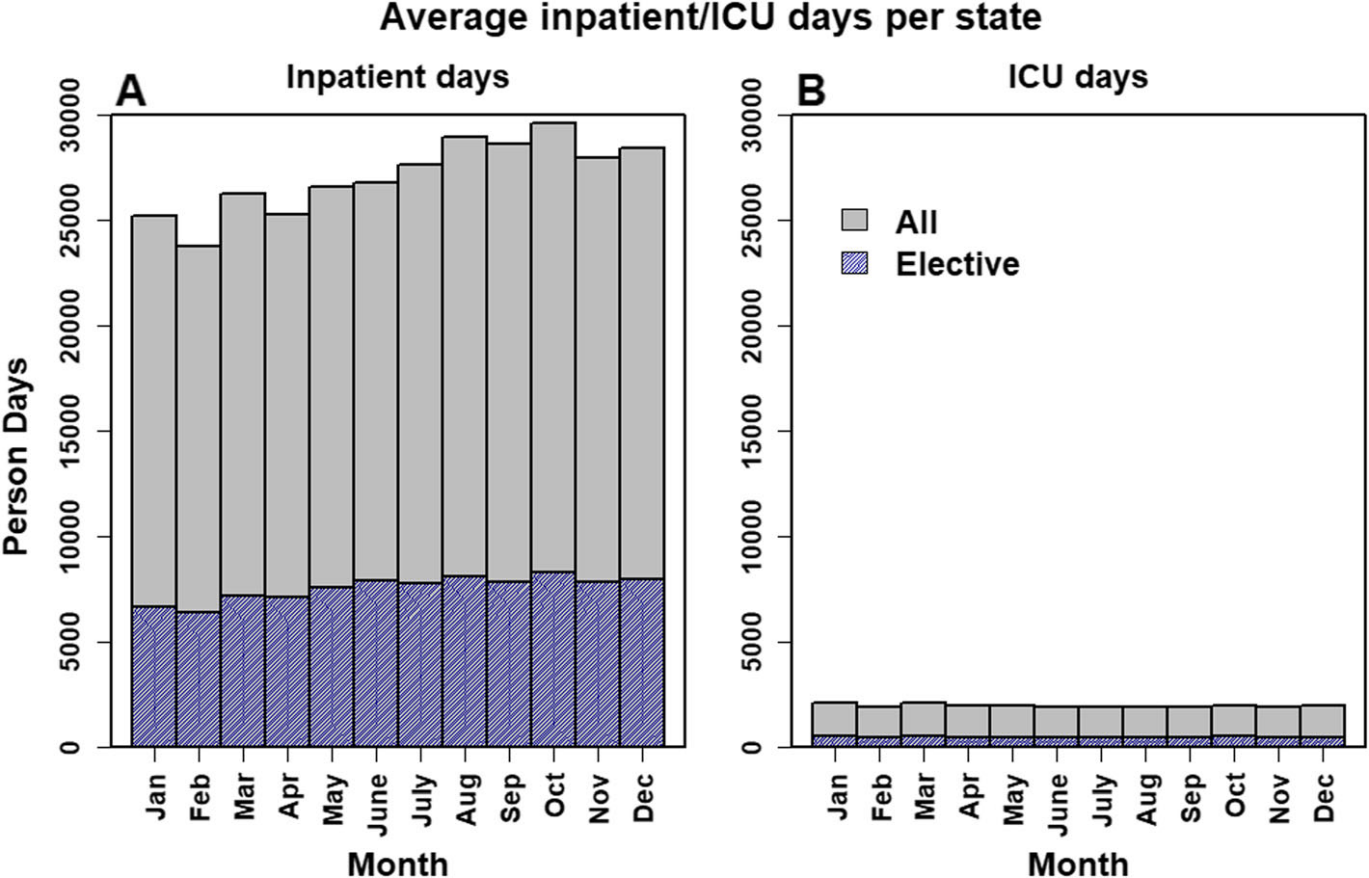
Average Proportion of Inpatient and ICU Person Days per State Resulting from Elective Surgery. Data from 161 million Marketscan patients from 2008 to 2017 displaying aggregated counts of hospital and ICU beds in total and those resulting from elective surgery averaged across all states. For each month

Across the U.S. there were an average of 1,442,013 (Interquartile range [IQR]: 1,378,039, 1,507,994) inpatient days per month and 104,265 ([IQR] 101,961, 104,842) ICU days per month in the MS sample. Roughly 30% of these days were associated with elective surgery (Fig. 1). We applied these percentages to US hospital data and classified beds as emergent, elective, and available. Among 735,996 hospital beds in the US, 351,369 were emergent, 136,264 were elective, and 248,363 were available.

### Financial impact

**Fig. 2:**
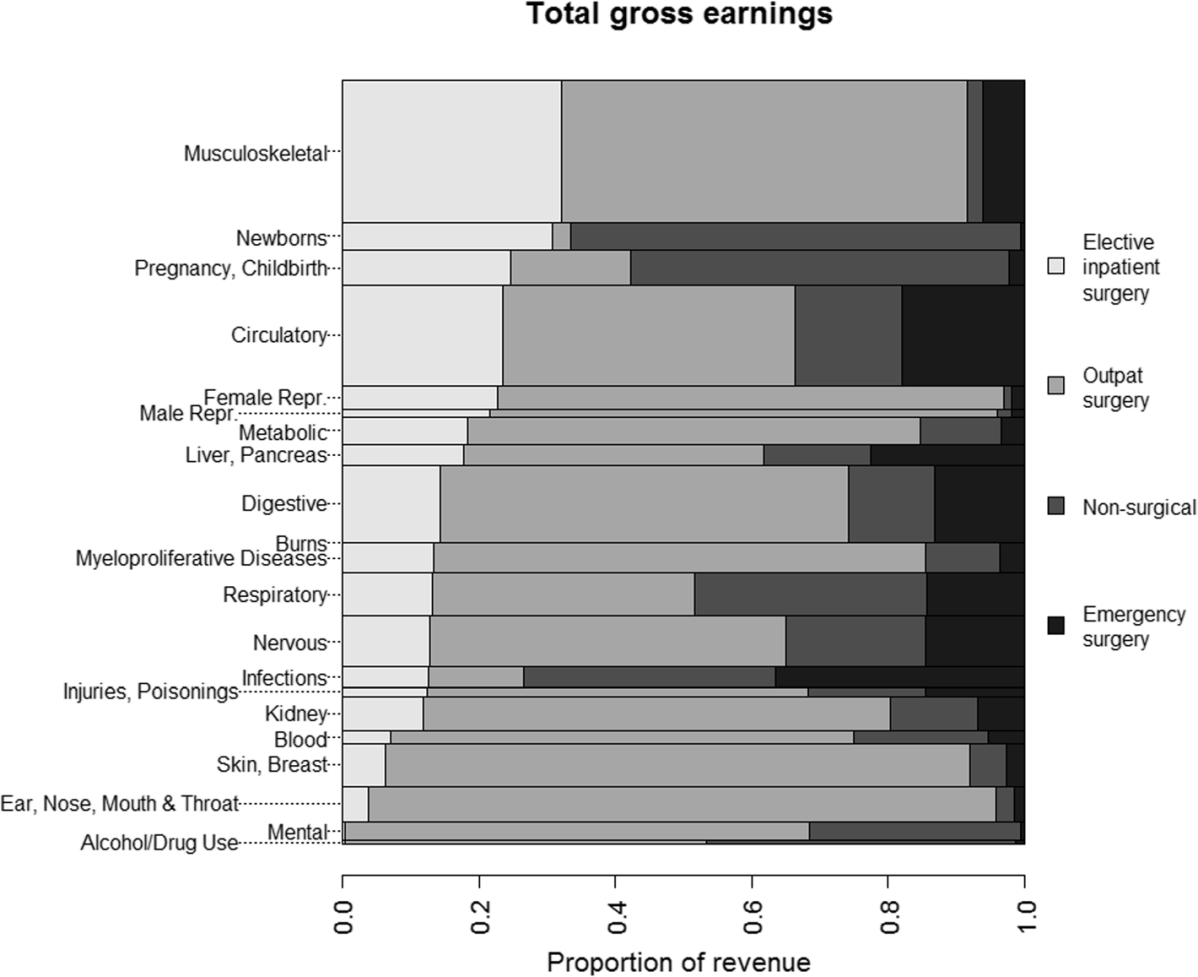
Financial contribution of major diagnostic categories (MDC) to gross hospital revenue. Data from 161 million Marketscan patients from 2008 to 2017 displaying aggregated gross hospital revenue by surgery type, separated by major diagnostic category (MDC), across the US. Levels are listed in descending order the percentage of each MDC category contributed by elective inpatient cases. Level width is proportional to the absolute value in US dollars

**Fig. 3:**
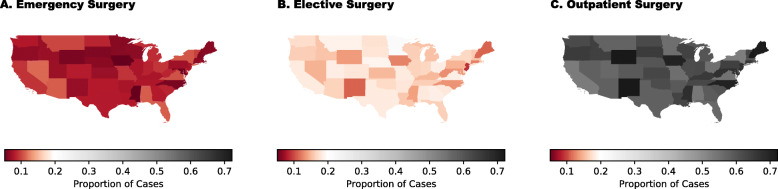
Regional variation by state in percentage financial contribution of non-elective, elective inpatient and outpatient surgeries. Panel **a** shows percent financial contribution to gross hospital revenue by state for non-elective cases. Panel **b** shows elective inpatient cases. Panel **c** shows outpatient cases

Elective surgical cases contribute 78% (IQR of statewide variation 74, 80) to of the gross inpatient and outpatient surgical revenue per year for hospitals represented in our sample, or 1.1 trillion US dollars. This value varies by MDC from $488 M to $231 billion (B) (median 39B; IQR 16B, 59B) (Fig. 2). Within each MDC, the percent of revenue from elective surgical cases varies from < 27 to 97% (median 68%; IQR 53, 86). When restricting our analyses to inpatient-derived revenue, elective surgery accounted for 43% (IQR of statewide variation 40, 45) of total gross revenue, or $254B. This value varies by MDC from < 1 to 88%, ($25 M to $80B). The relative financial contribution of elective surgical cases varies regionally and has important financial implications for hospital specific decisions on resumption of these elective procedures (Fig. 3).

### Case counts

With the cancellation of elective surgery, there is a need to balance revenue generation with capacity increases. Elective surgical procedures contribute 3B cases in the MS population, of which 7.9 M are inpatient. This varies by MDC (median 96 M [IQR 47 M, 224 M], range 1.9 M – 515 M), of which 197,396 (IQR 86781, 372,544; range 915–2.4 M) are inpatient. Elective musculoskeletal, circulatory and digestive categories comprise 1.1B cases, of which 3.8 M are inpatient. These three categories together compose 13% of hospital admissions, but 23% ($142B) of inpatient gross revenue. Including outpatient surgical cases, they compose 33% ($447B) of total inpatient and surgical-outpatient hospital revenue.

### Hospital and ICU capacity

Without cancelling elective surgery, median state hospital and ICU capacity was 4967 (IQR 1867, 6100) beds, and 743 (IQR 254.94, 944.37) respectively. This increased to 7692 (IQR 2553, 9679) hospital beds, and 991 (IQR 298, 1197) ICU beds if elective surgeries were cancelled.

**Fig. 4:**
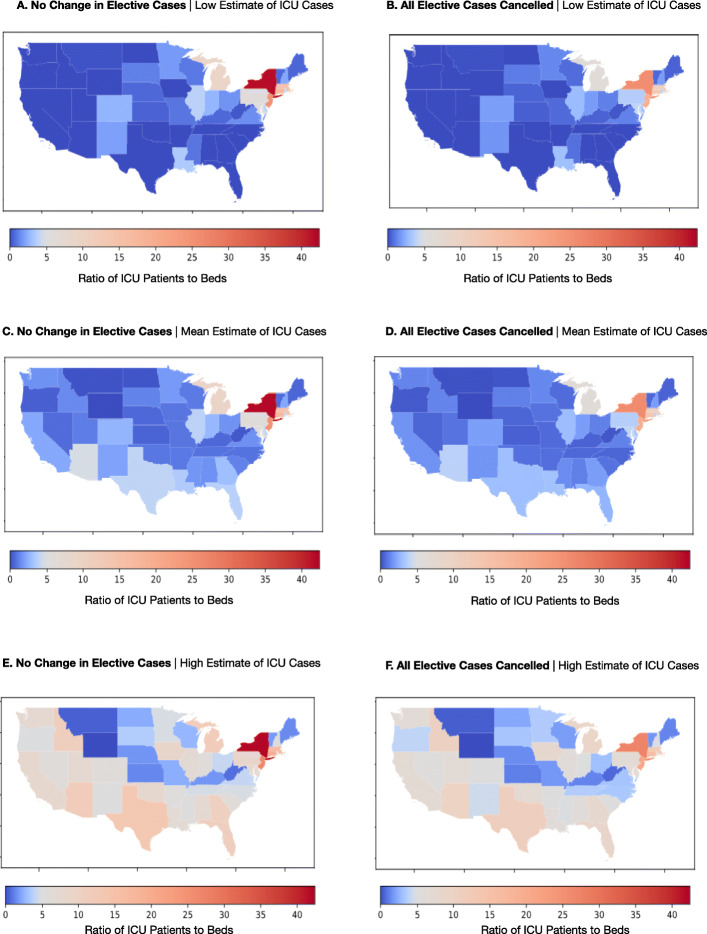
ICU Capacity across the US with and without cancelling elective surgeries. **a**-**f** Impact of Cancelling All Elective OR Cases on ICU Bed Availability if 5% of U.S. Population Infected with COVID-19. Estimates of low (**a**), mean (**c**) and high (**e**) IHME models. Additional capacity through cancellation of elective cases (**b**, **d**, **f**) was determined by applying estimates of the occupied and unoccupied beds resulting from elective surgery from the Marketscan database to the Harvard Global Health Institute (HGHI) estimates of total inpatient and ICU beds in each state

There is variability in hospital capacity by state (Fig. 4). As modeled, states would have mean 3.98 patients requiring admission per available ICU bed (Median 1.67; IQR: 0.89–3.79) (Fig. 4a, c). This distribution is highly skewed, with New York, New Jersey, Massachusetts, Michigan, and Pennsylvania having the highest patient to bed ratios. If mandates continue to be lifted, this increases to mean 5.25 patients per bed (Median 2.80; IQR: 1.59–5.53) (Fig. 4e). If Universal Mask wearing is followed in all public locations, states do not reach overcapacity levels on average. Removing elective surgeries will alleviate some of this stress (Fig. 4d), with LB estimates of an average of 2.37 ICU patients per bed IQR (0–1.98) (Fig. 4b) and UB estimates of an average 3.92 (IQR 1.29–4.03) (Fig. 4f).

## Discussion

Using billing and utilization data to model the financial contribution of elective surgical cases to hospital gross revenue, we demonstrate that cessation of elective musculoskeletal, cardiovascular and digestive cases account for the largest loss of hospital gross revenue, at $447B or 33% of all inpatient and surgical-outpatient revenue. In contrast, by case count, musculoskeletal, pregnancy, and circulatory categories account for the greatest contribution of elective inpatient cases. Applying case counts to the available hospital beds, cancelling elective surgeries at all US hospitals will decrease ICU overcapacity from 327 to 237% (lower bound) or from 525 to 392% (upper bound) assuming 5% of the U.S. population is infected as shown in our models, but at a financial cost. In light of the significant contribution of elective surgeries to gross hospital revenue, selective resumption of high contributing elective MDC categories, in patients who are COVID-19 negative, may be a way to resume surgery in a financially sustainable way when deemed safe. Outcomes from operative procedures in COVID-19 positive patients were recently demonstrated to be significantly worse, emphasizing the importance of careful patient selection and pre-operative COVID-19 testing for risk stratification in all patients, most especially for elective surgery [16].

Continued provision of care to patients with COVID-19 involves balancing increasing healthcare capacity with sustaining revenue generation. Previous models have addressed the expected healthcare burden of COVID-19 in various ways, but have not addressed revenue generation. To our knowledge, ours is the first to report case distribution and financial contribution of elective surgeries. We incorporated state specific billing data, reporting the relative contribution of elective surgeries to gross hospital revenue, and case counts, by MDC code. Our study informs the relative financial and capacity impact from selective resumption of elective cases. Further, we model the expected increase in bed capacity from cancelling these surgeries. Tsai et al previously estimated 1.7 excess COVID-19 patients per hospital bed and 4 excess patients per critical care bed assuming a 40% cumulative infection rate over 12 and 18 months, and an aggressive 50% bed availability [17]. Similarly, Murry used occupancy data from Medicare and Medicaid patients to estimate a capacity gap of 17,000 ICU beds and 64,000 hospital beds at peak infection assuming some measure of social distancing [18]. Our study differs from these by modeling disease transmission under a lower infection rate (5%) but faster course (100 days) than Tsai et al, and in a worse-case scenario without complete shelter in place orders, which 9 states do not currently have [19]. We also incorporated state-specific data for elective case volume and temporal variation. We demonstrated substantial state-level variation in overcapacity. This suggests an opportunity for regional cooperation and resource redistribution.

Our results are generated using a financial denominator of total inpatient revenue plus outpatient surgical cases, and do not account for the Medicare population that is not insured by Part C. We determined overcapacity values using consistent rates of infection across the population in less than 100 days and assumptions that regional rates are equal [6, 17, 18]. It is important to note that we applied rates of disease progression from regions prior to enforced distancing. Consistent with this, we assumed a stable rate of disease progression of 100 days since first case and do not account for policy responses to the pandemic, such as closure of non-essential service and stay-at-home orders, which are highly variable across states. It is important to note that patterns of gross hospital revenue and ICU bed use were based on the MS population which is not necessarily reflective of the U.S. population as a whole. In particular, patients included in MS carry private insurance through employers or employer-sponsored Medicare. Thus our cohort may be biased towards younger, healthier, and more affluent individuals and patterns of healthcare usage are likely to vary for the overall population. Our models assumed that all non-emergent cases would be postponed, however the degree to which a non-emergent procedure can be considered elective varies by patient age, comorbidities, etc. and our values may overestimate the proportion of surgeries that would truly be delayed.

## Conclusions

Elective inpatient surgeries account for 27% of hospital and ICU beds and 43% of gross revenue, which varies substantially by specialty. Among elective cases, musculoskeletal, cardiovascular and digestive MDC categories account for the largest contribution to hospital gross revenue, at 33%. The greatest contribution of bed capacity comes from musculoskeletal, pregnancy and circulatory categories. The cancellation of elective surgery will result in a substantial increase in hospital and ICU bed capacity, though this will vary between states, and at significant financial cost. If Universal Mask wearing is followed in all public locations, states do not reach overcapacity levels on average.

## Data Availability

To facilitate research reproducibility, replicability, accuracy and transparency, the associated analytic code is available on the Open Science Foundation [20] (OSF) repository, [DOI 10.17605/OSF.IO/U53M4] at [https://osf.io/u53m4]. The data that support the findings of this study were obtained under license from Truven. Data were received de-identified in accordance with Section 164.514 of the Health Insurance Portability and Accountability Act (HIPAA).

## Abbreviations

ACS: American College of Surgeons
B: Billion
CDC: Centers for Disease Control
COVID-19: Coronavirus Diseases 2019
DOI: Digital Object Identifier
HGHI: Harvard Global Health Institute
HIPAA: Health Insurance Portability and Accountability Act
ICU: Intensive care unit
IHME: Institute for Health Metrics and Evaluation
I/ICU: Inpatient and intensive care unit
LB: Lower bound
M: Million
MDC: Major diagnostic category
MMWR: Morbidity and Mortality Weekly Report
MS: Truven MarketScan
NHLBI: National Heart, Lung, And Blood Institute
NIH: National Institutes of Health
OSF: Open Science Foundation
STROBE: Strengthening the Reporting of Observational Studies in Epidemiology
UB: Upper bound
US: United States
